# Microbial Biosensor for Sensing and Treatment of Intestinal Inflammation

**DOI:** 10.1002/advs.202504364

**Published:** 2025-05-05

**Authors:** Duolong Zhu, Jeffrey Galley, Jason Pizzini, Elena Musteata, Martin V. Douglas, Walter J. Chazin, Eric P. Skaar, Jeffrey J. Tabor, Robert A. Britton

**Affiliations:** ^1^ Department of Molecular Virology and Microbiology Baylor College of Medicine Houston 77030 USA; ^2^ Systems, Synthetic, and Physical Biology Ph.D. Program Rice University Houston 77005 USA; ^3^ Department of Pathology Vanderbilt Institute for Infection Immunology, and Inflammation Microbiology, and Immunology Vanderbilt University Nashville 37235 USA; ^4^ Departments of Biochemistry and Chemistry Vanderbilt University Nashville 37235 USA; ^5^ Department of Bioengineering Department of Biosciences, Systems Synthetic, and Physical Biology Ph.D. Program Rice Synthetic Biology Institute Rice University Houston 77005 USA; ^6^ Department of Molecular Virology and Microbiology Alkek Center for Metagenomics and Microbiome Research Dan L. Duncan Comprehensive Cancer Center Baylor College of Medicine Houston 77030 USA

**Keywords:** biosensors, calprotectin, IL10, intestinal inflammation, Zur

## Abstract

Synthetic biology has enabled the development of biosensors to detect intestinal inflammation, yet few target the clinically validated biomarker of intestinal inflammation calprotectin with both diagnostic and therapeutic capabilities. Here, an optimized calprotectin biosensor is presented that leverages a zinc uptake regulator (Zur) controlled promoter coupled with a memory circuit to detect and record intestinal inflammation in vivo. The level of biosensor activation strongly correlates with calprotectin levels in the colon of two independent mouse models of colitis. Coupling of the biosensor with the production of the anti‐inflammatory cytokine IL10 allowed for the resolution of chemically induced colitis, demonstrating the ability of the biosensor to sense and respond to disease. This work highlights the utility of developing synthetic organisms for the diagnosis and treatment of intestinal disease using clinically validated biomarkers.

## Graphical abstract

The engineered calprotectin biosensor detects gut inflammation, processes the signal through genetic regulation, and responds by expressing the reporter sfGFP and the therapeutic protein secIL10. This dual functionality enables the engineered biosensor not only to sense but also to mitigate gut inflammation, highlighting its potential for disease‐responsive therapeutic intervention.

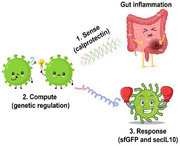

## 1 Introduction

The ability to monitor and manage chronic diseases is becoming more important as autoimmune diseases and diseases associated with aging are increasing worldwide. One of these disease areas, inflammatory bowel disease (IBD), affects more than 7 million people globally and is increasing at an alarming rate.^[^ ^1^ ^]^ IBD, which comprises Ulcerative Colitis and Crohn's Disease, has no known cure, requiring patients to manage symptoms throughout their lifetime. Predicting when patients are at risk of experiencing a flare of their disease is critical for the management of their symptoms, however, the current gold standard for assessing intestinal inflammation is endoscopy, which remains invasive, costly, and impractical for frequent monitoring. The ability of IBD patients in remission to have frequent feedback on their current inflammatory state in the gut would provide physicians the ability to catch potential flares before they exacerbate. Developing non‐invasive, real‐time tools to monitor gut inflammation will transform clinical outcomes by enabling early intervention.

Synthetic biology has emerged as a transformative field, offering the tools to engineer living systems for diverse applications, including disease diagnostics and therapeutics.^[^ ^2^, ^3^, ^4^ ^]^ Microbial biosensors for gastrointestinal (GI) disease monitoring, when administered orally, can be non‐invasive, safe, and inexpensive, while also providing necessary diagnostic information in rapid and real‐time conditions. The intestine is a logical site for employing engineered microbes as biosensors and for drug delivery as microbes have had a long evolutionary interaction with the human gut. Bacteria that have been developed to function as inflammation biosensors in animal models have been described. These biosensors target a variety of compounds including thiosulfate, tetrathionate, nitrate, pH, and reactive oxygen species, and are based on observations made primarily in mouse models of intestinal damage.^[^ ^5^, ^6^, ^7^, ^8^, ^9^, ^10^ ^]^ However, none of these compounds are currently biomarkers for human intestinal inflammation and their utility in clinical monitoring of intestinal inflammation remains to be tested.

Monitoring intestinal inflammation in patients in remission for IBD is challenging, as the most widely accepted non‐invasive test for gut inflammation relies on measuring fecal calprotectin concentration.^[^ ^11^, ^12^ ^]^ Calprotectin is expressed in various cell types, including monocytes, endothelial cells, and keratinocytes, and accounts for ≈60% of total soluble proteins in the cytosol fraction of neutrophils.^[^ ^13^, ^14^, ^15^ ^]^ Calprotectin functions as a heterodimeric form of the S100A8 and S100A9 EF‐hand Ca^2+^ binding proteins and sequesters essential transition metals (Zn^2+^, Mn^2+^, Fe^2+^) in the inflamed environment to restrict bacterial growth in a mechanism referred to as nutritional immunity.^[^ ^16^, ^17^, ^18^, ^19^ ^]^ While calprotectin is widely accepted as a biomarker for disease monitoring associated with Ulcerative Colitis, its utility in Crohn's Disease is less robust, particularly in small bowel disease.^[^ ^20^, ^21^ ^]^ Moreover, patient reluctance to collect their stool samples, along with the costs and delays associated with laboratory processing, further complicates timely diagnosis. By the time patients seek medical attention due to worsening symptoms, gut inflammation has often already progressed significantly. These challenges contribute to delays in both diagnosis and treatment, potentially exacerbating disease outcomes. Engineering a calprotectin‐responsive microbial biosensor capable of sensing intestinal inflammation is one potential way to address the IBD clinical need for non‐invasive diagnosis.

**Figure 1:**
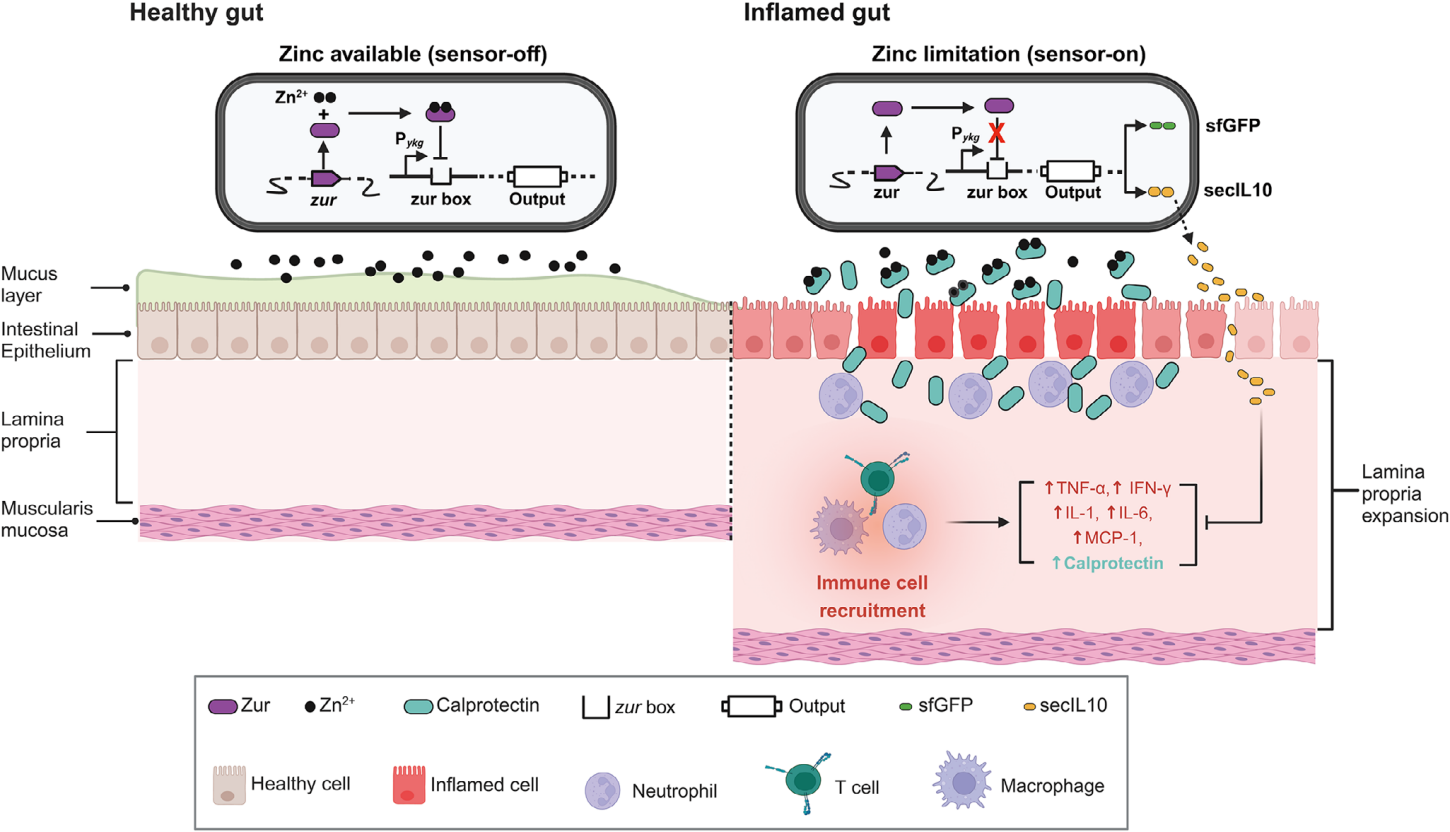
Summary of biosensor function in the healthy and inflamed intestine. The function of the bacterial biosensor in a healthy intestine (left) and an inflamed gut (right). In a healthy intestine, significant levels of zinc are present in the lumen, allowing for Zur to bind to zinc and actively repress the P*_ykg_* promoter. In the inflamed gut, immune cell infiltration and lamina propria expansion contribute to inflammation. Recruited neutrophils release calprotectin at the site of infection and sequesters zinc, reducing its bioavailability. This decrease in zinc levels leads to the release of the repressor Zur from the P*_ykg_* promoter, activating the engineered biosensor and triggering output expression. The activation of the biosensor (via the output of *sfgfp* expression) can be detected through qPCR and colony fluorescence imaging. For sense and respond activity, the production of secIL10 alleviates gut inflammation by inhibiting the production of pro‐inflammatory cytokines such as TNF‐α, IFN‐γ, IL‐1, and IL‐6 produced by immune cells.

We postulated that the human probiotic bacterium *Escherichia coli* Nissle (hereafter referred to as EcN) would be an ideal strain to develop an inflammation biosensor since it thrives in the inflamed gut, has had to evolve mechanisms for acquiring metals for growth in the presence of neutrophil infiltration, and is amenable to precision genome engineering.^[^ ^22^, ^23^ ^]^ Thus, we engineered a calprotectin‐responsive microbial biosensor capable of both detecting and recording gut inflammation (**Figure** **1**). To further expand the utility of this platform for therapeutic applications, we integrated a module for secretion of human IL10 carried by YebF^[^ ^24^ ^]^ (secIL10) to ameliorate intestinal inflammation. This engineering strategy enables our developed biosensor to deliver potent anti‐inflammatory cytokine directly to sites of inflammation, providing a dual diagnostic and therapeutic solution for managing intestinal inflammation. This work highlights the utility of developing synthetic organisms for the diagnosis and treatment of intestinal disease using clinically validated biomarkers.

## 2 Results

### 2.1 Identification of a Calprotectin Sensitive Promoter in *E. coli* Nissle

**Figure 2:**
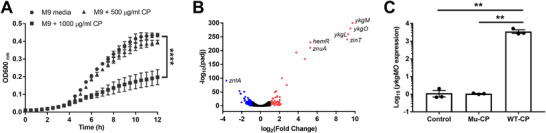
Identification of a calprotectin sensitive promoter in *E. coli* Nissle. (A) Growth profiles of EcN with calprotectin treated in M9 media. Data are mean ± SEM, *n* = 3 biological independent repeats. Statistical analysis was performed using a paired two‐tailed *t*‐test; ****p < 0.0001. (B) Volcano plot of significant (*padj* < 0.05) differentially expressed genes in EcN treated by wild‐type calprotectin (WT‐CP) and mutant calprotectin (Mu‐CP) in M9 media. Note: Since the *padj* values for *ykgM*, *ykgO*, *ykgL*, *zinT*, *hemR*, and *znuA* were calculated as 0, Prism GraphPad software assigned adjusted maximum values to display these genes in the figure. (C) Transcription of *ykgMO* detected by RT‐qPCR. EcN was treated with 500 µg mL^−1^ of WT‐CP, Mu‐CP, or calprotectin buffer for 30 min. Data are mean ± SEM, *n* = 3 biological independent repeats. Data were analyzed by using the comparative CT (2^−∆∆CT^) method. Statistical analysis was performed using one‐way ANOVA with the Tukey test; **p < 0.01.

To identify promoters in *E. coli* Nissle 1917 (EcN) that are upregulated upon exposure to calprotectin, we first measured the growth of EcN in the minimal media (M9) treated with two‐fold dilutions of calprotectin. We observed that EcN exhibited resistance to calprotectin concentrations up to 500 µg mL^−1^ (**Figure** **2**A). Subsequently, EcN was grown to mid‐log phase in M9 media and treated with 500 µg mL^−1^ of calprotectin for 30 min before RNA isolation and sequencing. A mutant calprotectin which lacks metal chelating activity was used as a negative control. We identified 61 upregulated and 52 downregulated genes in response to calprotectin, with the top three highly upregulated genes associated with the type B 50S ribosomal protein *ykgMO* operon (*ykqM*, *ykqO*, and *ykgL*) (Figure 2B and Table S1, Supporting Information). More than thousand‐fold of upregulation of *ykgMO* operon was further confirmed by RT‐qPCR (Figure 2C).

Given that calprotectin functions primarily as a high‐affinity chelator of metals such as zinc and manganese, it was unsurprising that the top‐upregulated genes were associated with metal starvation responses, especially for zinc. Bacteria use an evolutionarily conserved mechanism to regulate gene expression in response to zinc limitation that is dependent on the transcription factor Zur, which represses transcription in the presence of zinc‐replete concentrations and is inactive when intracellular zinc levels fall below a critical threshold.^[^ ^25^, ^26^ ^]^ In *E. coli*, Zur controls the expression of two ribosomal protein genes, *ykgM* and *ykgO*, that encode homologs of the ribosomal proteins L31 and L36, which require zinc for their function in the ribosome. These alternative ribosomal proteins do not require zinc for function, are not expressed under normal conditions, and are highly expressed upon zinc depletion to allow ribosome function when zinc concentrations are low.^[^ ^25^ ^]^ These properties of the *ykgMO* operon made it an attractive candidate to engineer for an inflammation biosensor.

### 2.2 Engineering of P*_ykg_* Regulation to Sense Physiologically Relevant Levels of Calprotectin

**Figure 3:**
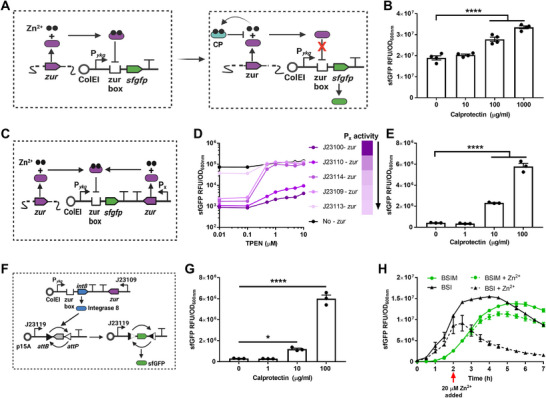
Engineering of P*_ykg_* regulation to sense calprotectin. (A) Schematic diagram of the biosensor with plasmid pBSI1 (PRB5000) responding to zinc limitation due to calprotectin. Left panel: zinc replete condition. Right panel: zinc depleted due to binding by calprotectin (CP). (B) Activation of biosensor PRB5000 treated with calprotectin in M9 media. Data are mean ± SEM, *n* = 3 biological independent repeats. Statistical analysis was performed using one‐way ANOVA with the Tukey test; ****p < 0.0001. (C) Schematic diagram for testing promoters to identify the amount of Zur required for the optimal dynamic range of the biosensor. P_x_ indicates where promoters of different strengths were integrated to alter the levels of *zur* in EcN. (D) Optimization of the biosensor by altering the level of *zur* expression. Promoters that were tested are indicated on the right with the strength of the promoter indicated by the color gradient. The darkest color indicates the strongest promoter with decreasing strength moving down. Each construct was incubated in M9 media supplemented with different amounts of TPEN (x‐axis) and fluorescence was measured and normalized to the density of the culture to account for any slight differences in cell numbers (y‐axis). Data are mean ± SD, *n* = 3 biological independent repeats. (E) Activation of biosensor BSI treated with calprotectin in M9 media. Data are mean ± SEM, *n* = 3 biological independent repeats. Statistical analysis was performed using one‐way ANOVA with the Tukey test; ****p < 0.0001. (F) Schematic diagram of memory circuit biosensor BSIM. A two‐plasmid system was created to allow for the biosensor to record that inflammation was detected. The first plasmid indicated at the top of the figure is the same as pBSI1 except the gene *int8* being placed under the control of P*_ykg_*. Expression of Integrase 8 leads to the inversion of the *sfgfp* gene in the second plasmid that allows for constitutive *sfgfp* expression. (G) Activation of biosensor BSIM treated with calprotectin in M9 media. Data are mean ± SEM, *n* = 3 biological independent repeats. Statistical analysis was performed using one‐way ANOVA with the Tukey test; *p < 0.05, ****p < 0.0001. (H) Stable expression of *sfgfp* in BSIM in Zn^2+^ replete conditions. BSI and BSIM were incubated in M9 medium with 100 µg mL^−1^ of wild‐type calprotectin to deplete zinc and induce biosensor activation. After 2 h, 20 µM of zinc was added to the indicated cultures and fluorescence was measured and normalized to OD_600nm_. Data are mean ± SEM, *n* = 3 biological independent repeats.

We first cloned the EcN *ykgMO* promoter (designated P*_ykg_*) upstream of the *sfgfp* gene in pColE plasmid (designated pBSI1) to monitor promoter activity in the presence of calprotectin (**Figure** **3**A). We incubated the resulting strain PRB5000 (EcN/pBSI1) with ten‐fold serial dilutions of calprotectin and observed a significant increase in *sfgfp* expression at calprotectin concentration exceeding 100 µg mL^−1^ (Figure 3B).

To enhance the dynamic range of the P*_ykg_* promoter‐based biosensor, we postulated that the high basal expression level was due to having P*_ykg_* on a multicopy plasmid, while *zur* was expressed in single copy on the chromosome. To address this problem, we constructed a series of plasmids with *zur* under the control of a range of promoters with different strengths (P_x_) on the same plasmid as the P*_ykg_*‐*sfgfp* construct (Figure 3C). Due to the limited availability of functional calprotectin protein, we instead used the metal chelator Tetrakis‐ (2‐pyridylmethyl) ethylenediamine (TPEN) to chelate zinc in the sensor optimization process. Among these constructs, we found the J23109 promoter driving *zur* expression was able to decrease basal expression from P*_ykg_* ≈100‐fold while retaining full induction by zinc limitation (Figure 3D; Figure S1, Supporting Information). The strongest promoters tested successfully repressed P*_ykg_* but did not allow full activation under zinc limitation while the weakest promoter had little effect on the basal level of transcription. We moved forward with J23109‐*zur* and designated this construct pBSI2 and the strain BSI (BioSensor of Inflammation). The optimized sensor BSI exhibited enhanced sensitivity for detecting calprotectin, with a detection threshold as low as 10 µg mL^−1^ compared to 100 µg mL^−1^ of PRB5000 in M9 media (Figure 3E).

While calprotectin is a well‐accepted biomarker for Ulcerative Colitis, there remains no generally accepted clinical biomarker for detecting inflammation non‐invasively in Crohn's Disease patients. Calprotectin likely fails to identify inflammation accurately in Crohn's patients due to the fact inflammation is patchy and often occurs in the small intestine, resulting in the loss of the calprotectin signal in feces.^[^ ^27^ ^]^ To address this, we developed a bacterial biosensor capable of both sensing and remembering inflammation (Figure 3F). We engineered a two‐plasmid system in which the first plasmid is identical to pBSI1 but included the gene integrase 8 (*int8*) driven by P*_ykg_* promoter (pBSIM1). On the second plasmid, the *sfgfp* gene is placed in the opposite orientation of the strong promoter J23119 and flanked by *attB* and *attP* sites, which are recognized by integrase 8 (pBSIM2). Expression of integrase 8 will flip the orientation of the *sfgfp* gene and allow for the constitutive expression of *sfgfp*. The EcN strain carrying both plasmids is referred to as BSIM (BioSensor of Inflammation with Memory).

BSIM was capable of activation at 10 µg mL^−1^ of calprotectin, similar to BSI (Figure 3G). To evaluate the dynamics of BSIM activation, we then compared the expression of *sfgfp* in BSI1 and BSIM after treatment with calprotectin or TPEN. BSI immediately showed induction of sfGFP that steadily increased over the course of the experiment (Figure 3H; Figure S2A,B, Supporting Information). For BSIM, expression of sfGFP was first observed 1.5–2 h after calprotectin or TPEN addition, likely due to the time needed to express Int8 and reverse the orientation of *sfgfp* within the population. To demonstrate the memory function of BSIM, we added 2–20 µM of zinc to the cultures after 2 h of induction. We found BSI showed an immediate decrease in activity after zinc addition, but BSIM continued to express sfGFP stably throughout the rest of the culture incubation and showed no difference in protein or RNA expression levels with or without the addition of zinc (Figure 3H; Figure S2C, Supporting Information). Additionally, we confirmed the biosensor BSIM was irreversibly activated by taking a subculture of the induced culture and incubating it in zinc‐replete conditions for multiple generations, observing the constitutive expression of sfGFP (Figure S2D–F, Supporting Information).

### 2.3 Detection of Calprotectin as an Intestinal Inflammation Biomarker by BSIM

**Figure 4:**
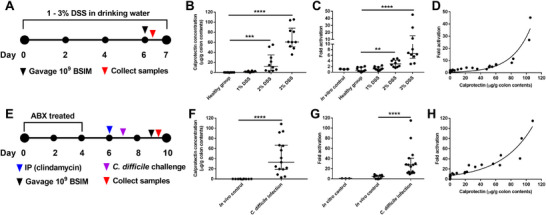
Detection of calprotectin as an intestinal inflammation biomarker by BSIM. (A) Experimental design for testing BSIM in DSS‐induced gut inflammation mouse model (five female and five male mice in each group). (B) Concentration of calprotectin in mouse colon contents. Colon contents at the end of the experiment were isolated and tested for calprotectin levels by ELISA. Data are median ± interquartile range, *n* = 10; Individual dots represent individual mice. Statistical analysis was performed using Kruskal–Wallis test; ***p < 0.001, ****p < 0.0001. (C) Fold activation of BSIM in mouse colon contents. Data are median ± interquartile range; for in vitro control *n* = 3 biological independent repeats, for in vivo experiments *n* = 10, individual dots represent individual mice. Statistical analysis was performed using Kruskal–Wallis test; **p < 0.01, ****p < 0.0001. (D) Correlation analysis of biosensor BSIM activation and colon calprotectin concentration. *n* = 40; individual dots represent individual mice. Correction analysis was performed by computing Pearson correlation coefficients with 95% confidence interval and two‐tailed *p*‐value analysis. (95% CI = 0.616–0.877, *r* = 0.778, *p* < 0.0001). (E) Experimental design for testing BSIM in *C. difficile* induced gut inflammation mouse model (for in vivo control group that treated with antibiotics but without *C. difficile* infection *n* = 10 with 5 female and 5 male mice, for *C. difficile* infection group *n* = 20 with ten female and ten male mice). (F) Concentration of calprotectin in the mice colon contents. Colon contents at the end of the experiment were isolated and tested for calprotectin levels by ELISA. Data are median ± interquartile range; Individual dots represent individual mice, for in vivo control group *n* = 10, for *C. difficile* infection group *n* = 15 (five mice died or difficult to get samples). Statistical analysis was performed using Mann–Whitney test; ****p < 0.0001. (G) Fold activation of BSIM in mouse colon contents. Data are median ± interquartile range; Individual dots represent individual mice, for in vivo control group *n* = 10, for *C. difficile* infection group *n* = 15 (five mice died or difficult to get samples). Statistical analysis was performed using Mann–Whitney test; ****p < 0.0001. (H) Correlation of biosensor BSIM activation and colon calprotectin concentration. *n* = 25; Individual dots represent individual mice. Correction analysis was performed by computing Pearson correlation coefficients with 95% confidence interval and two‐tailed *p* value analysis. (95% CI = 0.798–0.959, *r* = 0.907, *p* < 0.0001).

To demonstrate the utility of BSIM in accurately detecting gut inflammation, we tested the system in two independent mouse models of intestinal inflammation. We treated mice with 1–3% dextran sulfate sodium (DSS) for six days to induce inflammation and then orally delivered BSIM (**Figure** **4**A). After 4 h, the total DNA of colon contents and feces were isolated and tested by qPCR to identify the change in the flipped orientation of the *sfgfp* gene, indicating biosensor activation. Increasing concentrations of DSS resulted in increased levels of calprotectin in the colon contents (Figure 4B) and 3% DSS resulted in weight loss in the animals (Figure S3A, Supporting Information). BSIM was significantly activated in the mice treated with 2 or 3% DSS (Figure 4C) and the degree of activation strongly correlated with calprotectin levels in the intestine (Figure 4D, 95% CI = 0.616–0.877, *r* = 0.778, *p* < 0.0001). We also isolated several hundred colonies from each treatment and showed that a significantly higher proportion of isolated colonies had *sfgfp* activated in 3% DSS‐treated animals compared to healthy controls (Figure S3B, Supporting Information). Similarly, feces from 3% DSS‐treated mice also showed higher levels of calprotectin and BSIM activation compared to the healthy group (Figure S3C,D, Supporting Information). These results demonstrate that BSIM can accurately distinguish between healthy and inflamed mice in a calprotectin‐dependent manner.

To assess if inflamed mice feces can activate our biosensor directly in vitro, we incubated early log phase BSIM with 50% colon contents slurry (100 mg mL^−1^) containing the top three highest concentrations of calprotectin (112, 83, and 80 µg g^−1^ feces) in M9 media (Figure S4A, Supporting Information) or grew up BSIM with 10% colon contents slurry in M9 media (Figure S4B, Supporting Information). Our data showed that inflamed mouse fecal slurries cannot activate BSIM when incubated directly but could be activated when TPEN was added to the fecal slurry. This indicates that BSIM could have been activated in fecal slurries containing high levels of calprotectin and that the high concentration of zinc in feces limited biosensor activation (Figure S4C, Supporting Information).

To further demonstrate the ability of BSIM to sense inflammation in the intestine, we used a *Clostridioides difficile* infection model that induces a high level of neutrophil invasion and calprotectin release.^[^ ^28^ ^]^ Mice were treated with antibiotics and infected with R20291, a ribotype 027 strain of *C. difficile* that causes moderate to severe disease.^[^ ^29^ ^]^ After two days of *C. difficile* infection the mice were gavaged with BSIM and biosensor activation was monitored as described above (Figure 4E). We found that *C. difficile* infection caused a significant increase in calprotectin (Figure 4F) and activation of the BSIM occurred in mice infected with *C. difficile* (30‐fold increase) (Figure 4G). As observed in the DSS model, the level of activation had a strong correlation with the level of calprotectin detected in the colon (Figure 4H, 95% CI = 0.798–0.959, *r* = 0.907, *p* < 0.0001). These results indicate the BSIM system is accurately detecting levels of inflammation in the context of an inflamed gut in two independent models.

### 2.4 Calprotectin is Required for BSIM Activation In Vivo

**Figure 5:**
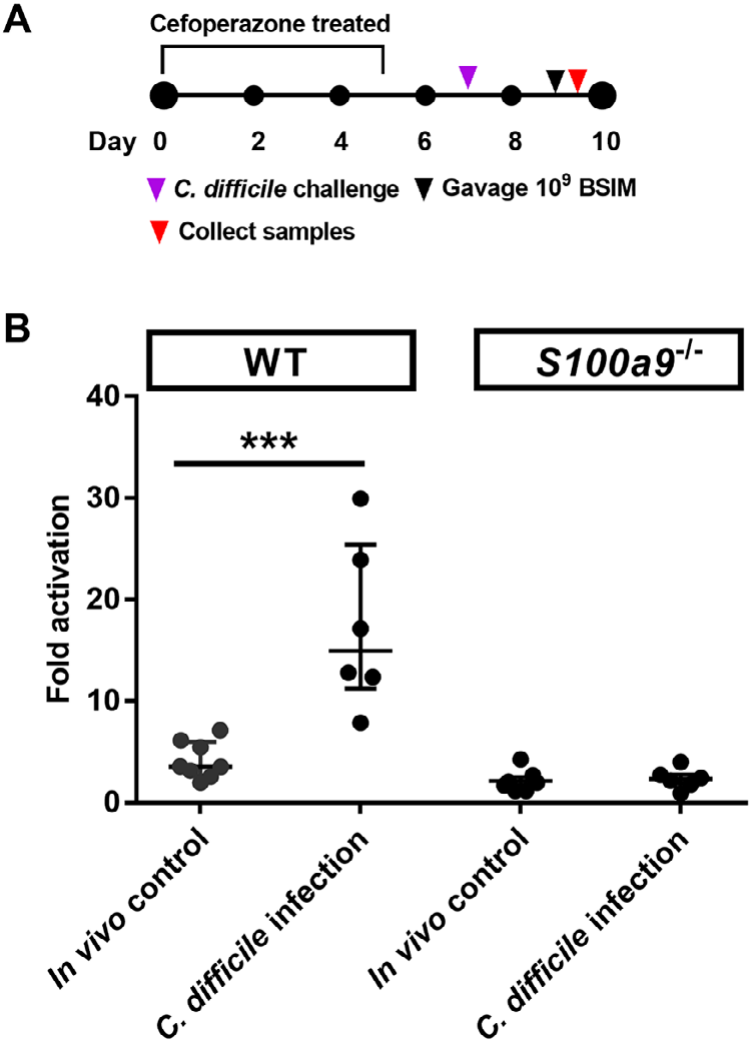
Calprotectin is required for BSIM activation in vivo. (A) Experimental design for testing BSIM in calprotectin deficient mice during *C. difficile* infection. (B) Fold activation of BSIM in mouse colon contents. Data are presented as median ± interquartile range. Individual dots represent individual mice. Wild type mice: in vivo control group, *n* = 10 (two samples with low quality of DNA were excluded), *C. difficile* infection group *n* = 10 (4 mice succumbed to infection or lacked intestinal contents); Calprotectin deficient mice (*S100a9* ^−/−^): in vivo control group *n* = 8 (one sample with low quality of DNA was excluded), *C. difficile* infection group *n* = 10 (four mice succumbed to infection or lacked intestinal contents). Statistical analysis was performed using Mann–Whitney test; ***p < 0.001.

To validate that calprotectin is essential for BSIM activation during inflammation in the intestine, we applied the biosensor BSIM to calprotectin‐deficient mice (*S100a9* ^−/−^) during *C. difficile* infection.^[^ ^17^, ^30^ ^]^ Mice were treated with the antibiotic cefoperazone and then infected with *C. difficile* CD196 spores. After two days of post‐infection, mice were gavaged with BSIM and euthanized 4 h later and colon contents were collected to measure BSIM activation (**Figure** **5**A). We observed that *C. difficile* infection in calprotectin‐deficient mice does not activate BSIM, whereas wild‐type mice infected with *C. difficile* infection exhibited significant activation of BSIM (Figure 5B). These results demonstrate that calprotectin is required for biosensor BSIM activation during intestinal inflammation.

### 2.5 Resolution of Colitis by Sensing and Responding to Intestinal Inflammation

**Figure 6:**
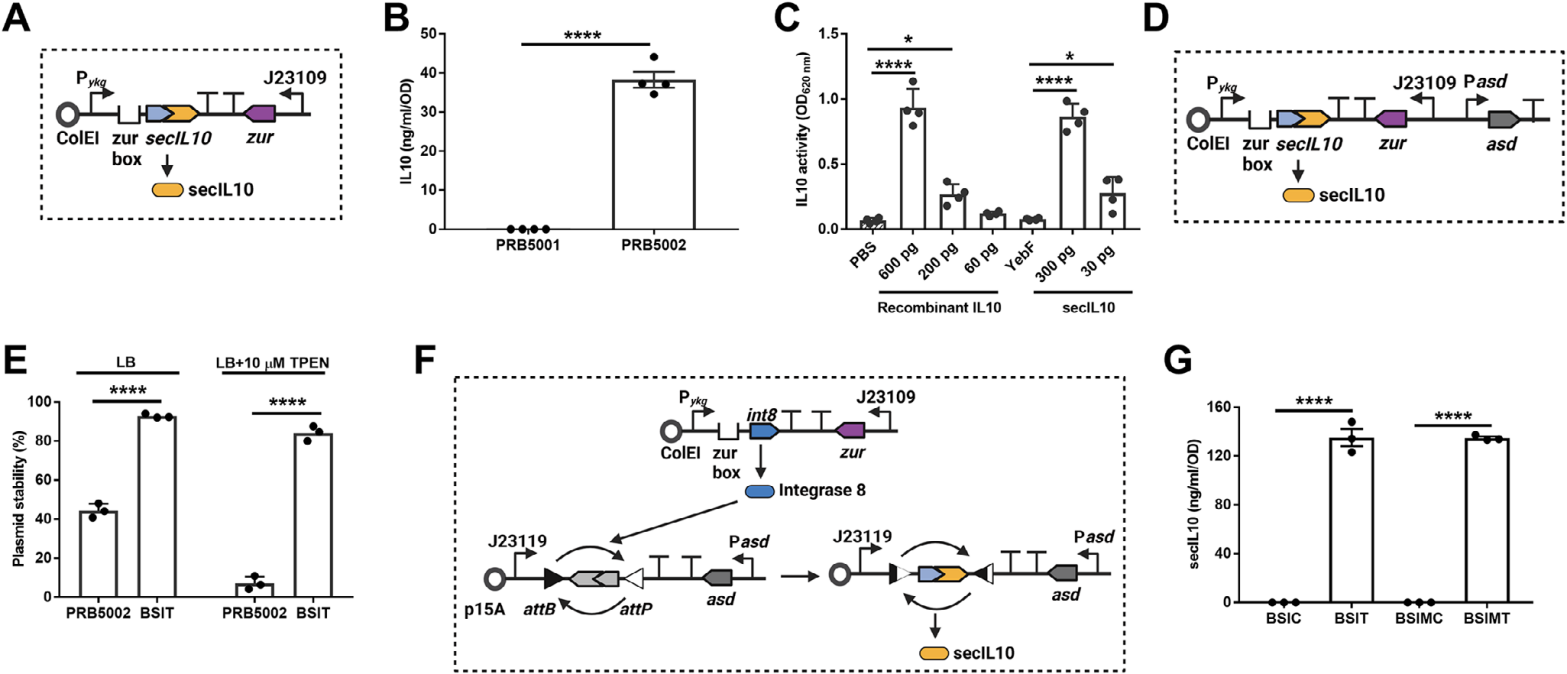
Engineering therapeutic biosensors to secret functional secIL10 (YebF‐IL10). (A) Diagram of therapeutic sensor plasmid pBSIDZ2. The *yebF* gene from EcN was fused into the N‐terminal of *IL10* (referred to as secIL10) and placed under the control of P*_ykg_* (secIL10 replaced *sfgfp* gene in pBSI2), resulting in therapeutic strain PRB5002. The *yebF* expression control strain PRB5001 was used as a control. (B) YebF facilitates secIL10 secretion in EcN. Biosensor PRB5002 (EcN/PBSIDZ2) was cultured to OD_600_ of 0.5–0.6 and then induced with 20 µM TPEN for 4 h in LB media. Following, the supernatants of cultures were collected and detected by IL10 ELISA kit. PRB5001 (EcN/PBSIDZ1) which expresses YebF only was used as a control. Data are mean ± SEM, *n* = 4 biological independent repeats. Statistical analysis was performed using an unpaired *t‐*test; ****p < 0.0001. (C) secIL10 exhibits functional IL10 activity in HEK‐Blue IL10 reporter cell line. Quantified secIL10 from PRB5002 supernatant was added to HEK‐Blue IL10 reporter cells line to activate reporter expression. Recombinant human IL10 was used as a positive control. Supernatants from PRB5001 (EcN/PBSIDZ1) with only YebF were used as a negative control. Data are mean ± SEM, *n* = 4 biological independent repeats; Statistical analysis was performed using ANOVA Tukey test; *p < 0.05, ****p < 0.0001. (D) Diagram of secIL10 stable expression sensor BSIT. The *asd* gene with its own promoter from EcN was cloned into pBSIDZ2 (referred to as pBSIT), following was transformed into EcNΔ*asd*, resulting in stable secIL10 expression therapeutic strain BSIT (EcNΔ*asd*/pBSIT). Control strain BSIC which only expresses YebF was used as a control. (E) BSIT exhibits plasmid stability in the absence of antibiotic selection. Therapeutic biosensors PRB5002 (EcN/pBSIDZ2) and BSIT (EcNΔ*asd*/pBSIT) were cultured in LB media or LB+10 µM TPEN without antibiotic selection, respectively. The biosensors were passaged five times, following 100 µL of ten‐fold serial dilution cultures were plated on LB agar plates, then a single colony was replica‐cultured in LB and LB with 15 µg mL^−1^ chloramphenicol agar plates to calculate plasmid stability. Data are mean ± SEM, *n* = 3 biological independent repeats. Statistical analysis was performed using an unpaired *t‐*test; ****p < 0.0001. (F) Diagram of therapeutic sensor plasmid (pBSIMT) with memory circuit. secIL10 replaced *sfgfp* gene in pBSIM2 with P*_asd_*‐*asd* (referred to as pBSIMT), following pBSIM1 and pBSIMT were transformed into EcNΔ*asd*, resulting in memory circuit therapeutic strain BSIMT. Control strain BSIC which only expresses YebF was used as a control. (G) Secretion of secIL10 in biosensor BSIT and BSIMT. Biosensors were cultured to OD_600_ of 0.5–0.6 and then induced with 20 µM TPEN for 4 h in LB media. The supernatants of cultures were collected and detected by IL10 ELISA. The expression of secIL10 in BSI1 and BSIM remains similar, indicating no impact on IL10 expression with the addition of *asd* to the plasmid. Data are mean ± SEM, *n* = 3 biological independent repeats. Statistical analysis was performed using one‐way ANOVA with the Tukey test; ****p < 0.0001.

Microbial biosensors have been engineered and adapted to self‐tunable expressing therapeutic agents for in situ and real‐time IBD therapy.^[^ ^3^, ^31^, ^32^, ^33^ ^]^ IL10 (interleukin 10) as an anti‐inflammatory cytokine has shown beneficial effects in ameliorating gastrointestinal tract inflammation both in mice and humans.^[^ ^34^, ^35^, ^36^, ^37^ ^]^ To ask if our bacterial biosensors could be re‐designed to sense inflammation and deliver an anti‐inflammatory therapeutic in response, we replaced the *sfgfp* gene in pBSI2 either with YebF protein (which has been shown to facilitate the secretion of proteins in *E. coli*)^[^ ^24^ ^]^ or the human IL10 gene fused to the YebF (referred to hereafter as secIL10) resulting in the control strain PRB5001 (EcN/pBSIDZ1) and the therapeutic sensor PRB5002 (EcN/pBSIDZ2) (**Figure** **6**A). Induction of P*_ykg_* with TPEN yielded ≈38 ng mL^−1^ per OD of secIL10 into the extracellular media after 4 h of induction in LB media (Figure 6B). We next confirmed that secIL10 is functional using the Human & Murine IL10 reporter cells (InvivoGen, San Diego, CA) that couples IL10 receptor activation to a colorimetric reporter output. In comparison to recombinant IL10, secIL10 displayed a similar level of IL10 activity, indicating the fusion is fully functional (Figure 6C).

Because plasmids will not be under selection while in the mouse intestine, we engineered the expression of the *asd* gene into the system, which encodes the enzyme aspartate semialdehyde dehydrogenase required for lysine, threonine, and methionine biosynthesis (Figure 6D). Disruption of *asd* in the *E. coli* Nissle chromosome (∆*asd*) makes the growth of the strain‐dependent on the retention of the plasmid with *asd*.^[^ ^9^ ^]^ We confirmed that the addition of *asd* to pBSIDZ2 (pBSIT) resulted in significant retention of the plasmid in the absence of selection (Figure 6E) as previously described.^[^ ^9^ ^]^ We refer to this system as BSIT (BioSensor of Inflammation with Therapeutic). We made the same modifications to the BSIM plasmid constructs resulting in BSIMT (BioSensor of Inflammation with Memory and Therapeutic) (Figure 6F) and validated a similar level of secIL10 production (Figure 6G).

**Figure 7:**
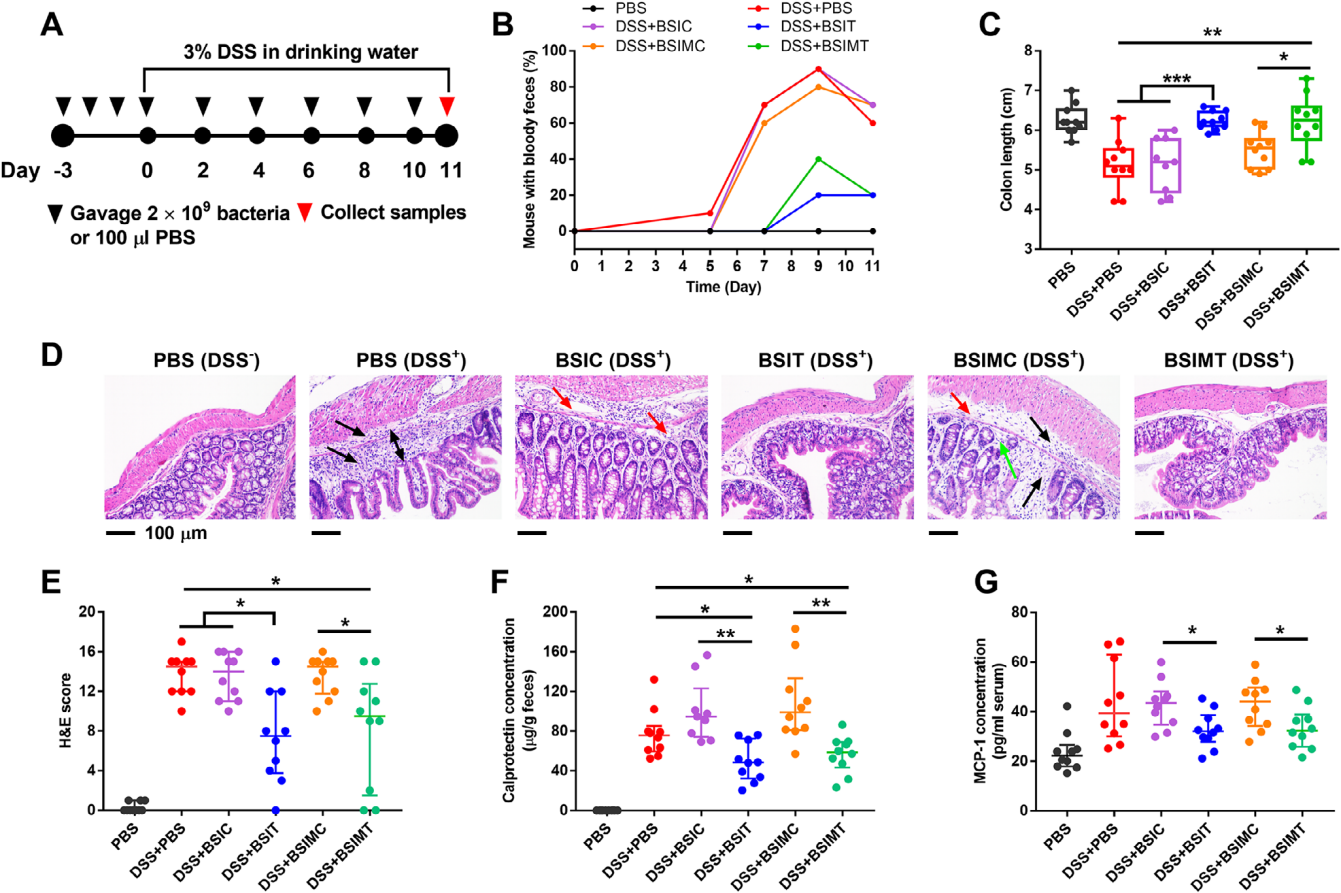
Biosensor coupling with secIL10 ameliorates intestinal inflammation. (A) Experimental design of therapeutic biosensors applied to 3% DSS‐induced gut inflammation for the treatment of intestinal inflammation. Each group has ten male mice. (B) Percentage of mice with bloody feces. Mice producing bloody feces were monitored and recorded every day for 11 days. Groups tested include DSS + BSIT, DSS + BSIMT, DSS + BSIC (control expressing only YebF), DSS + BSIMC (control expressing only YebF), and DSS + PBS (colitis control). Groups were compared to animals that received only gavage of PBS without DSS treatment. (C) Colon length. Data are presented in a box & whiskers plot with min to max, showing all points, *n* = 10, and individual dots represent individual mice. Statistical analysis was performed using Kruskal–Wallis test; *p < 0.05, **p < 0.01, ***p < 0.001. (D) Histological score of colon tissue. Mouse distal colon tissue was collected, fixed, sliced, stained (H&E staining), and scored. Data are median ± interquartile range, *n* = 10; Individual dots represent individual mice. Statistical analysis was performed using Kruskal–Wallis test; *p < 0.05. (E) Representative hematoxylin and eosin (H&E) stained images of mouse distal colon sections from each experimental group. Black arrows: inflammatory cell infiltrates in the mucosa and submucosa. Double‐headed black arrow: lamina propria expansion. Red arrows: loose of connective tissue. Green arrows: disappearance of glands and goblet cells. (F) Concentration of calprotectin in mice feces. Mice fecal samples were collected on day 11 and tested for calprotectin concentration by ELISA. Data are median ± interquartile range, *n* = 10; Individual dots represent individual mice. Statistical analysis was performed using Kruskal–Wallis test; *p < 0.05. (G) Concentration of MCP‐1 (Monocyte Chemoattractant Protein‐1) in mice serum. Mice serums were collected on day 11 and tested for MCP‐1 concentration by ELISA. Data are median ± interquartile range, *n* = 10, Individual dots represent individual mice. Statistical analysis was performed using Kruskal–Wallis test; *p < 0.05.

To test if BSIT and BSIMT could sense, respond, and alleviate intestinal inflammation, we treated mice with 3% DSS along with BSIT, BSIMT, and controls of *E. coli* Nissle only expressing YebF (designated BSIC and BSIMC). We gavaged animals with each strain for three days prior to starting DSS treatment and then provided the organisms every other day during 3% DSS treatment (**Figure** **7**A). Animals receiving only PBS vehicle were used as an additional negative control. We found that BSIT and BSIMT were able to significantly improve several parameters associated with DSS colitis including reduction in mice with bloody feces (Figure 7B), improving colon length (Figure 7C), improved histological scores (Figure 7D,E) and weight loss (Figure S5, Supporting Information) compared to the PBS, BSIC, and BSIMC treated controls. We also found that BSIT and BSIMT treatment resulted in reduced levels of calprotectin in the feces (Figure 7F) and reduced MCP‐1 (Monocyte Chemoattractant Protein‐1) in the serum (Figure 7G). These data show that both systems can sense intestinal inflammation and resolve inflammation by production of an anti‐inflammatory protein.

To compare our sensing and responding system with continuous secIL10 expression, we replaced the P*_ykg_* promoter in pBSIT with constitutive promoters of varying strengths. Constitutive expression with medium‐to‐strong promoters slowed bacterial growth, while a weak promoter had no impact but failed to produce detectable secIL10. The strain PRB5004, using the J23115 promoter, achieved comparable secIL10 production to BSIT while only causing a slight growth delay (Figure S6A,B, Supporting Information). In a 3% DSS‐treated mouse model, BSIT significantly improved histological scores and colon length, whereas PRB5004 did not show the same efficacy (Figure S6C–F, Supporting Information). BSIT‐treated mice also exhibited higher serum secIL10 levels than those treated with PRB5004 (Figure S6G, Supporting Information). As many immunologic treatments for IBD and other inflammatory diseases are delivered systemically and have unwanted side effects associated with body‐wide immunosuppression, the ability to sense and respond to disease and regulate expression of therapeutics to only occur when disease is encountered may alleviate side effects associated with systemic therapeutic delivery.

## 3 Discussion

In this report, we demonstrate the optimization of a naturally occurring ribosomal promoter gene circuit (P*_ykg_*) that can accurately detect intestinal inflammation in two independent models of intestinal inflammation. The level of activation of BSIM in both the DSS and *C. difficile* infection models showed a strong correlation between the amount of calprotectin in the intestine and level of activation of the biosensor. We also demonstrated that calprotectin is required for activation of the biosensor in vivo, indicating that the levels of zinc present in the inflamed gut are sufficient to suppress BSIM activation in the absence of calprotectin. Therefore, while the biosensor directly senses the levels of zinc bioavailable to the bacterium, BSIM functions as an indirect biosensor of calprotectin in the gut. The strong correlation between colonic calprotectin levels and biosensor activation in colitis indicates that rather than being an all‐or‐nothing response there is the ability to sense different levels of inflammation. Our data from the DSS and *C. difficile* colitis models demonstrates that our engineering approach to P*_ykg_* generated a biosensor that can accurately respond to different levels of calprotectin.

An important finding of this work is that the P*_ykg_* promoter is naturally tuned to respond to the levels of zinc limitation^[^ ^38^, ^39^, ^40^ ^]^ that occur during neutrophil infiltration and inflammation in the gut. We posited this would be the case as *E. coli* has co‐evolved with humans for millennia and the system was evolutionarily optimized to respond to nutritional immunity. While gut zinc depletion can occur due to non‐inflammatory factors such as dietary inhibitors, genetic variations, hormonal influences, and altered microbiota,^[^ ^41^, ^42^ ^]^ we anticipate that our engineered sensor activates only when zinc levels drop to the extremely low threshold to render Zur inactive, and this likely occurs only at sites of inflammation. Further studies are needed to identify potential off‐target conditions unrelated to gut inflammation. Consistent with the idea that sensor activation would occur only at sites of inflammation, we did not observe activation of the biosensor when incubated with mouse colon contents containing high levels of calprotectin (Figure S4A,B, Supporting Information). This is most likely that despite high levels of calprotectin in the colon contents, there is still enough free zinc available to suppress BSIM (Figure S4C, Supporting Information), as supported by the activation of BSIM after the addition of a metal chelator. This contrasts with a recent report that identified the same promoter, P*_ykg_*, as a calprotectin‐sensitive promoter and was able to show activation of their promoter in fecal samples of IBD patients.^[^ ^43^ ^]^ Differences in the engineering of the P*_ykg_* genetic circuit or the long incubation times (12 h) needed to activate their biosensor in human feces may contribute to the different responses we see with our constructs. Nonetheless, the independent identification of P*_ykg_* to detect intestinal inflammation by two independent groups highlights the utility of using microbial biosensors in clinically managing IBD and other intestinal diseases. Our work further extends the utility of this biosensor to both sense and respond to disease, resulting in resolution of inflammation.

Our memory circuit biosensor is a prototype for detecting the patchy inflammation that occurs in the small intestine in Crohn's Disease, where diseased tissue is flanked by healthy tissue. One hurdle that needs to be overcome is the ability to measure the level of activation in situ without the need for the handling of feces. Coupling biosensor output with a visible color change in stool or with devices capable of transmitting real‐time signals from the gut directly could significantly enhance our biosensor applications. Recently, a device that can couple the sensing of nitrate as a proxy for intestinal inflammation and transmit this information from the gut was reported,^[^ ^44^ ^]^ suggesting in situ detection and transmission of inflammation signal using our engineered biosensors is an achievable goal. Currently, while our memory circuit biosensor can detect and retain inflammation signals in Crohn's Disease, it cannot distinguish Crohn's Disease from Ulcerative Colitis. Engineering biosensors to respond to more specific Crohn's Disease biomarkers, such as fecal lactoferrin and calgranulin C (S100A12), could enhance diagnostic precision and broaden sensor applications. Additionally, multi‐sensor systems capable of detecting multiple biomarkers may further improve sensor specificity, though potentially at the cost of reduced sensitivity.

In our therapeutic studies, constitutive expression of secIL10 in *E. coli* Nissle showed limitations, as high levels of secIL10 secretion impaired bacterial growth and failed to resolve disease in the DSS colitis model (Figure S6, Supporting Information). This highlights the need for tightly regulated therapeutic expression to balance efficacy and bacterial fitness. By coupling biosensor activation to the production of secIL10, our system delivers localized therapeutic intervention only at sites of inflammation and could ameliorate the gut inflammation. Since many immunologic treatments for IBD and other inflammatory diseases are delivered systemically and cause unwanted side effects due to widespread immunosuppression, a disease‐responsive approach that regulates therapeutic expression only when disease is present may help mitigate these side effects. Our work highlights the transformative potential of synthetic biology to develop integrated diagnostic‐therapeutic platforms, paving the way for more effective management of chronic inflammatory diseases.

## 4 Conclusion 

We developed and optimized a calprotectin‐responsive biosensor capable of detecting and responding to intestinal inflammation by leveraging the zinc uptake regulator (Zur) and a memory circuit. Our findings demonstrate that the biosensor accurately detects inflammation associated with calprotectin levels in vivo and can be further engineered to produce the anti‐inflammatory cytokine IL10 for therapeutic intervention. This dual‐function biosensor provides a promising approach for non‐invasive diagnosis and treatment of inflammatory bowel diseases.

## 5 Experimental Section

### Bacterial Strains and Media

All strains used are listed in Table S1 (Supporting Information). *Escherichia coli* Nissle 1917 (referred hereafter as EcN) was grown aerobically at 37 °C using either Luria‐Bertani (LB) broth (1% tryptone, 0.5% yeast extract, 1% NaCl) or the minimal defined media M9 (200 mL 5×M9 salts+0.4% glucose+2 mM MgSO_4_+0.1 mM CaCl_2_+0.2% casamino acids for 1 liter M9 media). *Escherichia coli* DH5α was used as a cloning host. Chloramphenicol (15 µg mL^−1^) and ampicillin (100 µg mL^−1^) were used for *E. coli* selection when needed. *Clostridioides difficile* R20291 was cultured in BHIS media (brain heart infusion broth supplemented with 0.5% yeast extract and 0.1% L‐cysteine, and 1.5% agar for agar plates) at 37 °C in an anaerobic chamber (90% N_2_, 5% H_2_, 5% CO_2_). For spores preparation, *C. difficile* strains were cultured in Clospore media and purified as described earlier.^[^ ^45^ ^]^ All chemicals were purchased from Sigma‐Aldrich (St. Louis, US), unless otherwise specified.

### Minimum Inhibitory Concentration (MIC) Assay

EcN was cultured to log phase in M9 media, following, 10^4^ CFU of bacterial cells treated with two‐fold dilutions of calprotectin (calprotectin buffer: 20 mM Tris, 100 mM NaCl, 3 mM CaCl_2_) in 200 µL of fresh M9 media were seeded into a 96 well plate. OD_600nm_ value was measured every 30 min for 12 h by a TECAN plate reader. Recombinant human calprotectin was supplied by Dr. Walter Chazin of Vanderbilt University.

### Transcriptome Analysis

A single colony of EcN was grown overnight in M9 media, then diluted 1:100 into 5 mL of fresh M9 media and cultured to log phase. EcN cultures were subsequently treated with 500 µg mL^−1^ of either wild‐type calprotectin or mutant calprotectin (lost metal binding activity) for 30 min. Transcription was halted by adding RNA*later*™ solution (Invitrogen, USA), followed by cell collection via centrifugation. RNA was extracted using the QIAGEN RNeasy kit (Qiagen, Hilden, Germany) according to the manufacturer's protocol. The isolated RNA samples were frozen and sent to Azenta (Burlington, MA, USA) for RNA sequencing and data analysis. Raw counts were obtained, and gene expression analysis was conducted using DESeq2, with a false discovery rate (FDR) adjusted *p* value cutoff of 0.05 for significance between wild‐type and mutant calprotectin‐treated groups.

### Construction and Optimization of Biosensor Plasmids

The *ykgMO* (paralog for L31/L36 ribosomal accessory protein) promoter was cloned directly upstream of a super fold green fluorescent protein cassette (*sfgfp*) in pColE1 plasmid via the Gibson Reaction (New England Biosciences, Ipswich, Massachusetts). The resulted plasmid and biosensor strain were named as pBSI1 (pColE1‐P*ykg*‐ *sfgfp*) and PRB5000, respectively.

To reduce the background of the primary P*_ykg_* biosensor, the zinc‐binding transcription factor *zur* gene was inserted into PRB5000 under the control of different constitutive expression promoters. Among them, five different constitutive promoters J23100, J23110, J23114, J23109, and J23113 from the Registry of Standard Biological Parts (http://parts.igem.org/Promoters/Catalog/Anderson) were selected and tested. The sensitivity and expression strength of new biosensor constructs was analyzed through fluorescence detection.

Because it was expected that zinc sequestration to levels that will activate the biosensor will only occur at sites of active inflammation with neutrophil infiltration, it was posited that the ideal biosensor would need to become permanently activated upon sensing inflammation for future readout in the stool. A memory switch biosensor was developed containing a two‐plasmid system using phage integrase 8 being driven by the P*_ykg_* promoter regulated by Zur on one plasmid (pBSIM1) and a *sfgfp* gene on the second plasmid in the opposite orientation of the strong promoter J23119 (denoted pBSIM2). The *sfgfp* gene was flanked by *att* sites that were recognized by the integrase and when expressed will flip the orientation of the *sfgfp* gene to allow expression from promoter J23119. All Gibson oligos were made using IDT, and all amplicons were synthesized with Phusion polymerase (NEBiosciences, Ipswich, Massachusetts). A listing of all constructed plasmids and primers used in this study can be found in Table S2 and Table S3 (Supporting Information).

### Calprotectin and TPEN Induction and Metal Complementation

Recombinant human calprotecin (WT‐CP) and the multi‐site mutant lacking transition metal binding capacity (Mu‐CP) were produced as described previously.^[^ ^46^, ^47^ ^]^ A two‐fold dilution series of recombinant human calprotectin (1–500 µg mL^−1^) and TPEN (*N*,*N*,*N′*,*N′*‐tetrakis(2‐pyridinylmethyl)‐1,2‐ethanediamine) (1.5–30 µM) were used to assess the induction of biosensor constructs. EcN cultures were grown to log phase in M9 media and subsequently treated with calprotectin or TPEN in a 96‐well plate, with a final volume of 200 µL per well. The plate was incubated at 37 °C in a Tecan plate reader, and fluorescence and OD600_nm_ values were measured every 30 min for 12 h.

To evaluate the stability of the memory circuit biosensor, biosensor cultures were first induced with either calprotectin or TPEN for 2 h, followed by the addition of varying concentrations of zinc chloride (ZnCl₂). The cultures were incubated for an additional 12 h under the same conditions. Fluorescence and OD600_nm_ values were measured every 30 min for 12 h using the Tecan plate reader.

### Evaluation of Biosensor Activation In Vivo

Six‐week‐old C57BL/6 mice were procured from Baylor College of Medicine (Houston, Texas, USA) and transferred to an established protocol room that was approved by the Baylor College of Medicine Institutional Animal Care and Use Committee (IACUC). All mice were housed in 12:12 light: dark light cycles at room temperatures ranging between 22 °C and 24 °C and humidities between 40% and 60% in an SPF clean room. Five mice were housed in one cage with autoclaved Biofresh pelleted cellulose bedding and fed with regular LabDiet 5V5R chow supplied by Baylor College of Medicine animal facility.

For dextran sodium sulfate (DSS, MW = 40,000, Thermos Scientific) induced IBD mouse model, 6‐week‐old C57BL/6 mice (five female and five male mice in each group) were treated with or without 1–3% (w/v) DSS in drinking water for seven days. On the sixth day, mice were gavaged with 10^9^ CFU of the memory circuit biosensor.^[^ ^5^ ^]^ After 4 h, mice were euthanized by CO_2_ and colon contents were collected for the next analysis.

To test biosensor in the *Clostridioides difficile* infection mouse model (CDI), six‐week‐old C57BL/6 mice (for the healthy group *n* = 10 with 5 female and 5 male mice, for the *C. difficile* infection group *n* = 20 with 10 female and 10 male mice) were given an orally administered antibiotic cocktail (kanamycin 0.4 mg mL^−1^, gentamicin 0.035 mg mL^−1^, colistin 0.042 mg mL^−1^, metronidazole 0.215 mg mL^−1^, and vancomycin 0.045 mg mL^−1^) in drinking water for 4 days. After 4 days of antibiotic treatment, all mice were given autoclaved water for 2 days, followed by one dose of clindamycin (10 mg kg^−1^, intraperitoneal route) 24 h before the spores challenge (Day 0).^[^ ^48^ ^]^ After that, mice were orally gavaged with 10^4–5^ of R20291 spores or PBS as a control. 2 days after spores gavage, 10^9^ CFU of memory circuit biosensor were gavaged to all mice. 4 h after biosensor gavage, colon contents were collected for biosensor activation test.

### Test of Biosensor in Calprotectin Deficient Mouse Model

To confirm calprotectin was the key factor of our biosensor activation, BSIM biosensor was applied to the calprotectin‐deficient mice with *C. difficile* infection model. The animal experiments under protocol M2300018 were reviewed and approved by the Institutional Animal Care and Use Committee of Vanderbilt University. Procedures were performed according to the institutional policies, Animal Welfare Act, NIH guidelines, and American Veterinary Medical Association guidelines on euthanasia. C57BL/6 male mice (7 weeks old; Jackson Laboratories) or *S100a9* ^−/−^ (7 weeks old; bred in‐house) were housed in groups of five and maintained at Vanderbilt University Medical Center Animal Facilities.

Mice were treated with 0.5 mg mL^−1^ cefoperazone in drinking water for 5 days, followed by 2 days of recovery with normal drinking water.^[^ ^30^ ^]^ Mice were gavaged with 10^5^ of CD196 spores in 100 µL of PBS. Before infection, mice were confirmed to be *C. difficile* negative via plating. After 48 h postinfection, mice were gavaged with 10^9^ CFU of biosensor BSIM and euthanized 4 h later with colon contents and feces collected.

### Detection of Biosensor Activation by qPCR

The total DNA from colon contents was isolated by E.Z.N.A Stool DNA kit (Omega) for biosensor activation analysis by qPCR with primers T‐F/T‐R. Primers T‐F/T‐R can only detect the flipped *sfgfp* gene (sensor on). When *sfgfp* was on the reverse orientation (sensor off status), T‐F/T‐R primers cannot amplify the PCR bands. Data were analyzed by using the comparative CT (2^−∆∆CT^) method with ampicillin resistance gene (*amp*) in pBSIM2 as a control. Fold increase in the flipped orientation of *sfgfp* compared to the in vitro control was presented.

### Construction of Therapeutic Biosensor

To engineer human IL10 secretion strain, facilitating carrier protein YebF was fused up to IL10 and assembled into plasmid pBSI2 which replaced *sfgfp*, respectively. The corresponding therapeutic strain was denoted as PRB5002 (with pBSIDZ2 plasmid) and the *yebF* expression strain PRB5001 (with pBSIDZ1 plasmid) was used as a control.

To achieve stable expression of secIL10, an essential gene *asd* that was required for lysine, threonine, and methionine biosynthesis in EcN was deleted by CRISPR‐Cas9^[^ ^49^ ^]^ and complemented the *asd* gene in sensor plasmid pBSIT, resulting a therapeutic sensor BSIT. To construct the continuous secIL10 expression strain, the P*_ykg_* promoter in pBSIT was replaced with constitutive promoters of varying strengths. Meanwhile, the *sfgfp* gene in pBSIM2 was replaced with secIL10, and the *asd* gene was assembled into pBSIM2 to get the stable therapeutic biosensor with a memory circuit (BSIMT). Strains with only *yebF* expression plasmid (pBSIC and pBSIMC) were used as controls (BSIC and BSIMC). Supernatants of therapeutic biosensor cultures with TPEN induced were analyzed by IL10 ELISA kit (EAGLE Biosciences, Nashua, NH). The activity of secIL10 was analyzed by the Human&Murine IL‐10 reporter cells (HEK‐Blue^TM^ IL10 Cells) that was engineered to respond to functional IL‐10 according to the product instruction (InvivoGen, San Diego, CA).

### Evaluation of Therapeutic Biosensors on Gut Inflammation In Vivo

To evaluate the therapeutic biosensors for inflammation amelioration in vivo, the DSS‐induced gut inflammation animal model was used. Six‐week‐old C57BL/6 male mice were orally gavaged 2 × 10^9^ therapeutic sensor bacteria or 100 µL PBS for 3 days, following mice were treated with or without 3% DSS for 11 days. 2 × 10^9^ therapeutic sensor bacteria or 100 µL PBS were gavaged every 2 days. One group of mice gavaged with PBS without DSS treatment was used as a control.

The mice weight and disease severity were monitored every day. On day 11, mice fecal pellets were collected first, then the mice were euthanized following blood serum, colon, and colon contents were collected for analysis. MCP‐1 concentration in blood serum was measured by MCP‐1/CCL2 mouse ELISA kit (Thermo Fisher Scientific Inc, USA). Mouse distal colon tissue was fixed, sliced, and stained with hematoxylin & eosin (H&E staining). A blinded histological scoring was performed. Six inflammation‐related items (inflammatory infiltrate, goblet cell loss, crypt hyperplasia, muscle thickening, submucosal inflammation, ulceration) were evaluated with each item from 0 to 3 score based on disease severity.^[^ ^50^ ^]^

### Statistical Analysis

Statistical analyses and graphical data visualization were conducted using GraphPad Prism 7. Data are presented as means ± SEM or median ± interquartile range, as specified in each figure legend according to the experimental design. Sample size (*n*) for each statistical analysis was noted in the corresponding figure legend. Statistical significance was defined as *p < 0.05.

## Conflict of Interest

R.A.B. and D.Z. have submitted a patent application based on this work. R.A.B. and J.D.G. have submitted a patent application based on this work. R.A.B. and J.J.T. are co‐founders of PanaBio and served on the S.A.B. and R.A.B. is a co‐founder of Mikrovia and is on the S.A.B. of Tenza. All other authors declare they have no competing interests.

## Author Contributions

R.A.B. conceptualized the study. D.Z., J.D.G., J.P., E.M., J.J.T., R.A.B., W.J.C., E.P.S., and M.V.D. developed the methodology. D.Z., J.D.G., and M.V.D. conducted the investigation. J.J.T. and R.A.B. supervised the work. D.Z., J.D.G., J.J.T., R.A.B., M.V.D., and E.P.S. wrote the original draft. All authors reviewed and edited the manuscript.

## Ethical Statement

All animal experiments and procedures were approved by Baylor College of Medicine (protocol: AN‐7117 and AN‐6675) and Vanderbilt University (protocol: M2300018‐00) Institutional Animal Care and Use Committees (IACUC).

## Supporting information



Supporting Information

Supplemental Table 1

## Data Availability

The data that support the findings of this study are available from the corresponding author upon reasonable request.
