# Supplementary material for: Microbial Biosensor for Sensing and Treatment of Intestinal Inflammation
Source: Adv Sci (Weinh). 2025 May 5;12(27):2504364. doi: 10.1002/advs.202504364 (PMC12279244; doi:10.1002/advs.202504364)
Supplement: Supplementary file 1 — Supporting Information [file ADVS-12-2504364-s001.docx]

**Supporting Information for**

**Microbial biosensor for sensing and treatment of intestinal inflammation**

*Duolong Zhu^*^, Jeffrey Galley, Jason Pizzini, Elena Musteata, Martin V. Douglas, Walter J. Chazin, Eric P. Skaar, Jeffrey J. Tabor and Robert A. Britton^*^*

Corresponding author: [robert.britton@bcm.edu](mailto:robert.britton@bcm.edu), duolong.zhu@bcm.edu.

**Supporting Information**

Supporting Figures 1 to 6

Supporting Tables 2 to 3

**Supporting Figures**


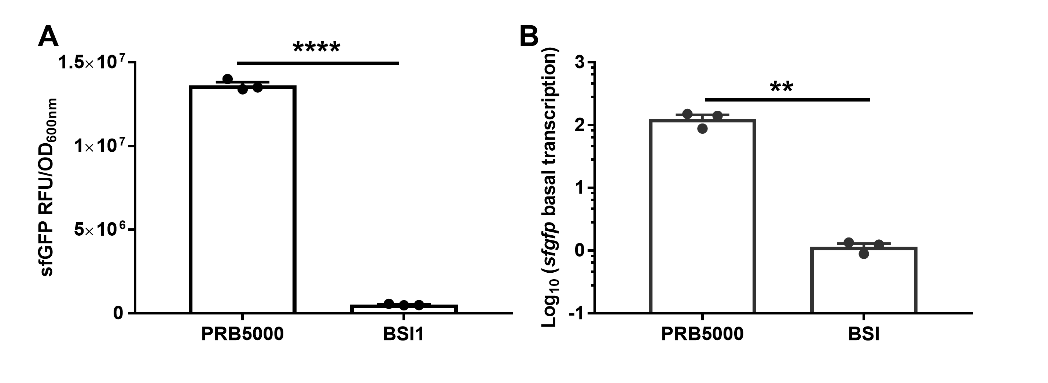


**Figure S1. Stable expression of *sfgfp* in memory circuit biosensor BSIM.**

**(A)** Basal expression of *sfgfp* in biosensor PRB5000 and BSI. PRB5000 and BSI were cultured in M9 media without calprotectin addition for 12 h. Following, fluorescence was detected and normalized to OD600_nm_. Data are mean ± SEM, n = 3 biological independent repeats. Statistical analysis was performed using an unpaired *t* test; *****p* < 0.0001.

**(B)** Basal transcription of *sfgfp* in biosensor PRB5000 and BSI detected by RT-qPCR. PRB5000 and BSI biosensors were cultured for 6 h, then total RNA was isolated for *sfgfp* gene transcription analysis. Data are mean ± SEM, n = 3 biological independent repeats. Statistical analysis was performed using an unpaired *t* test; ***p* < 0.01.


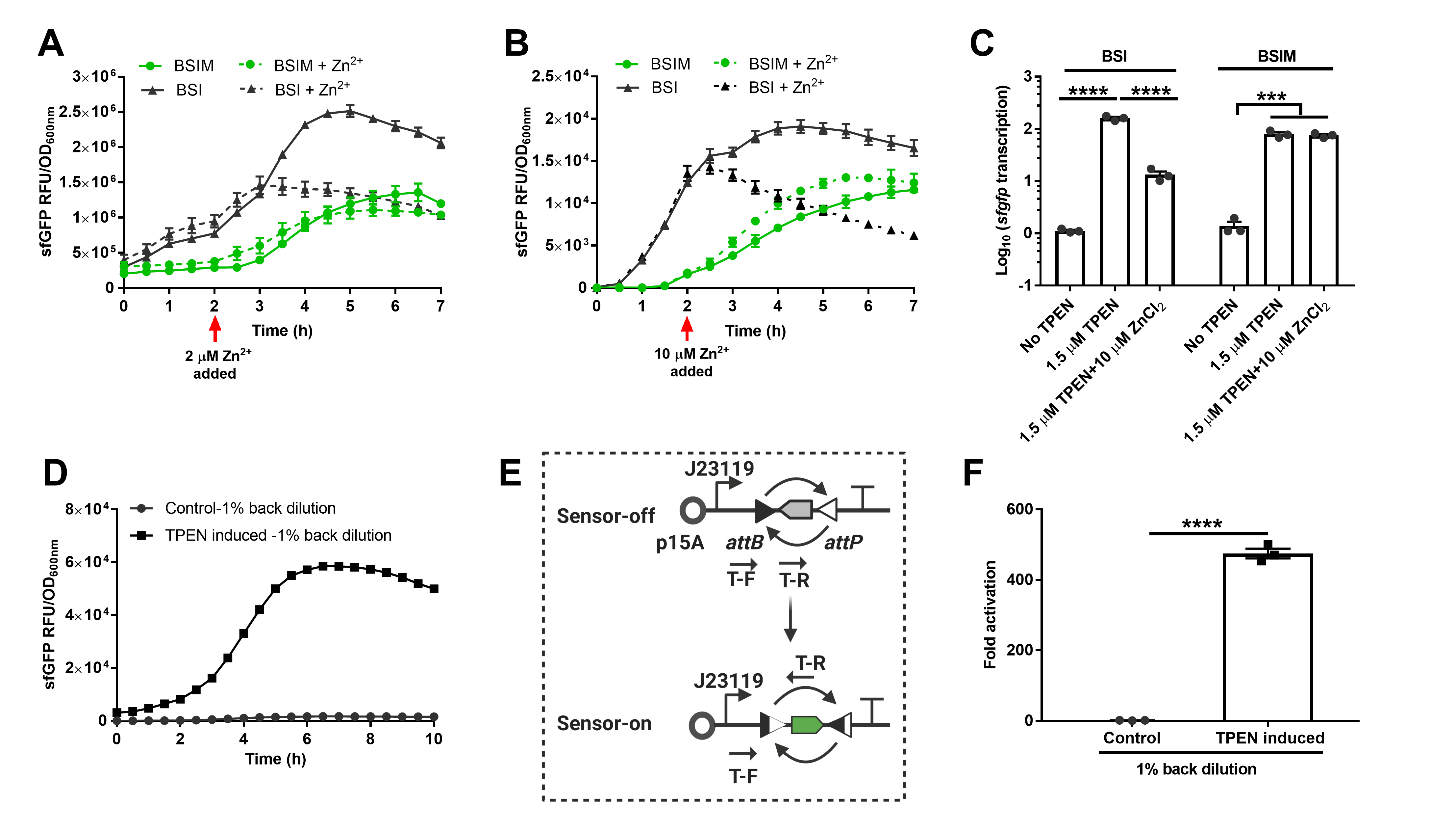


**Figure S2. Stable expression of *sfgfp* in memory circuit biosensor BSIM.**

**(A)** Stable expression of *sfgfp* in BSIM pre-induced by calprotectin in Zn^2+^ replete conditions. BSI and BSIM were cultured in M9 medium with 10 µg mL^-1^ of wild type calprotectin to deplete zinc and induce biosensor activation. After 2 hours, 2 µM of zinc was added to the indicated cultures and fluorescence was measured and normalized to OD_600nm_. Data are mean ± SEM, n = 3 biological independent repeats.

**(B)** Stable expression of *sfgfp* in BSIM pre-induced by TPEN in Zn^2+^ replete conditions. BSI and BSIM were cultured in M9 medium with 1.5 µM TPEN to deplete zinc and induce biosensor activation. After 2 hours, 10 µM of zinc was added to the indicated cultures and fluorescence was measured and normalized to OD_600nm_. Data are mean ± SEM, n = 3 biological independent repeats.

**(C)** Transcription of *sfgfp* in BSI and BSIM after Zn^2+^ addition. BSI and BSIM were cultured in M9 medium with 1.5 µM TPEN to deplete zinc and induce biosensor activation. After 2 hours, 10 µM of zinc was added. Total RNA from BSI and BSIM cultures was isolated four hours after zinc addition. Transcriptional levels of *sfgfp* were measured by RT-qPCR. Data are mean ± SEM, n = 3 biological independent repeats. Statistical analysis was performed using ANOVA Tukey test; ****p* < 0.001, *****p* < 0.0001.

**(D)** The BSIM biosensor was induced with 1.5 µM TPEN for 4 h, then 1% of cultures were subcultured into fresh M9 media without TPEN. Fluorescence was monitored every 30 min for 10 hours. BISM without TPEN induction was used as a control. Dada showed here was from the fifth passage. Data are mean ± SEM, n = 3 biological independent repeats.

**(E)** Schematic diagram of BSIM sensor off and sensor on. Primers T-F/T-R were used for the flipped *sfgfp* gene detection. When *sfgfp* is on the reverse orientation (sensor off status), T-F/T-R primers can’t amplify the PCR bands.

**(F)** Fold change of flipped *sfgfp* (fold activation) in 1% back dilution cultures at 8 h growth. Plasmids from 1% back dilution cultures were isolated and used for qPCR test with primers T-F/T-R. The ampicillin resistance gene (*amp*) on the sensor plasmid pBSIM2 was used as the reference gene. Data are mean ± SEM, n = 3 biological independent repeats. Statistical analysis was performed using an unpaired t test; *****p* < 0.0001.


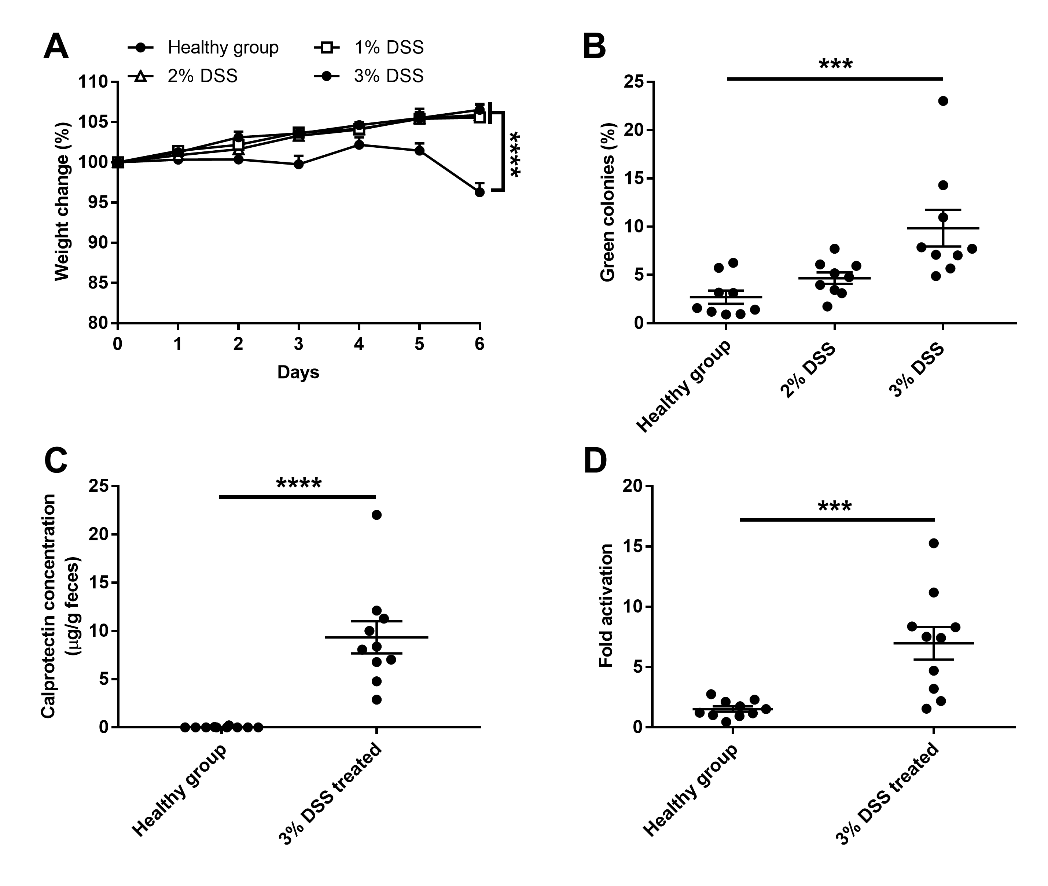


**Figure S3. Detection of calprotectin as an intestinal inflammation biomarker by BSIM.**

**(A)** Weight change of mice treated with 1-3% DSS. Data are mean ± SEM, n = 10 for each group, individual dots represent individual mice. Statistical analysis was performed using a paired *t* test; *****p* < 0.0001.

**(B)** Percent of bacterial colonies showing BSIM biosensor activation (green colonies) in the mouse colon detected by plating. Colon contents from healthy, 2%, and 3% DSS treated groups were serially diluted in PBS and plated on LB agar plates with 15 µg mL^-1^ chloramphenicol and 100 µg mL^-1^ ampicillin to select cells harboring BSIM. Colonies were analyzed by fluorescence microscopy and the percentage of green colonies per total colonies is reported. Data are mean ± SEM**,** n = 9, individual dots represent individual mice. Statistical analysis was performed using Kruskal-Wallis test; ****p* < 0.001.

**(C)** Concentration of calprotectin in 3% DSS treated mouse feces. Feces of 3% DSS treated mice at the end of the experiment were collected and tested for calprotectin levels by ELISA. Data are median ± interquartile range, n = 10, individual dots represent individual mice. Statistical analysis was performed using Kruskal-Wallis test; *****P* < 0.0001.

**(D)** Fold activation of BSIM in 3% DSS treated mouse feces. Data are median ± interquartile range, n = 10, individual dots represent individual mice. Statistical analysis was performed using Kruskal-Wallis test; ****p* < 0.001.


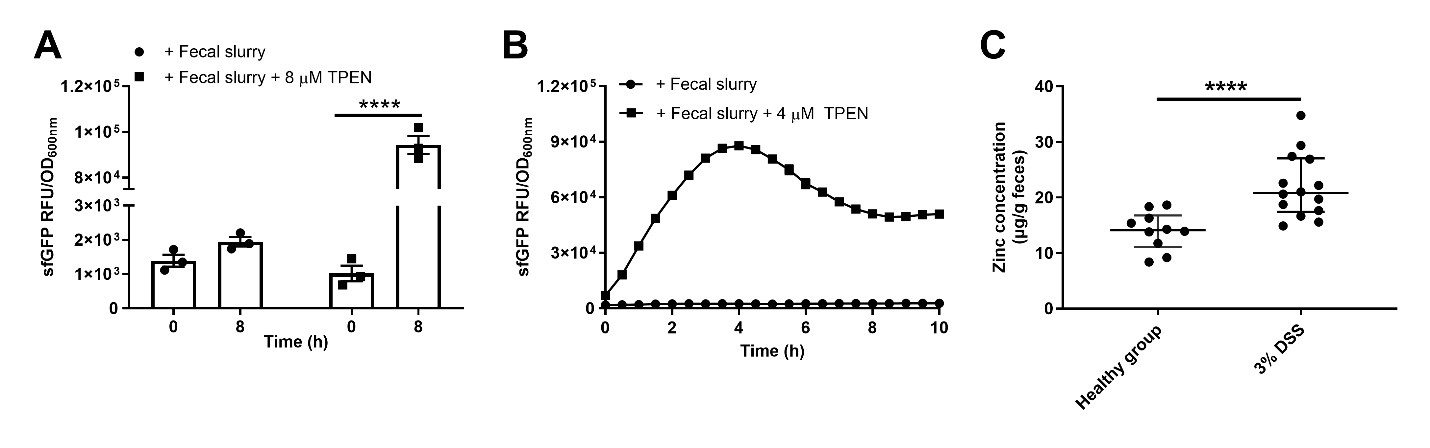


**Figure S4. Colon contents with high concentration of calprotectin from inflamed mice cannot activate BSIM *in vitro*.**

**(A)** BSIM activation with 100 mg mL^-1^ 50% colon contents slurry *in vitro*. BSIM were cultured to the early logarithmic growth phase. Subsequently, colon contents slurry (100 mg mL^-1^) containing the three highest concentrations of calprotectin (112, 83, and 80 µg g^-1^ feces) was added to the BSIM cultures for 8 h, respectively. To demonstrate that BSIM can be activated in the presence of these samples, colon contents slurries were treated with 8 µM TPEN was tested. Data are presented as mean ± SEM**,** n = 3, individual dots represent individual mice fecal slurry. Statistical analysis was performed using an unpaired t test; *****p* < 0.0001.

**(B)** Fluorescence of BSIM cultured with 10% colon contents slurry in M9 media. BSIM (OD_600nm_ = 0.1) was cocultured with the colon contents slurry with the highest concentration of calprotectin (112 µg g^-1^ feces). To demonstrate BSIM can be activated by metal sequestration in slurries, the colon contents slurry with 4 µM TPEN was tested. Fluorescence was monitored every 30 min for 10 hours. Data are mean ± SEM, n = 3 biological independent repeats.

**(C)** Zinc concentration in feces. Zinc levels in fecal samples from healthy and inflamed (treated with 3% DSS) mice were measured using a colorimetric zinc assay kit (Novus Biologicals, NBP324548) according to the instruction. Feces were resuspended in Milli-Q water at a final concentration of 50 mg mL^-1^ and processed for zinc quantification. Data are presented as median ± interquartile range. Each dot represents an individual mouse (healthy control group: n = 10; DSS treated group: n = 14). Statistical significance was performed using Mann–Whitney test; *****p* < 0.0001. Note: Fecal samples were from the same animals shown in Figure 7, specifically from the PBS and PBS+DSS treated groups.


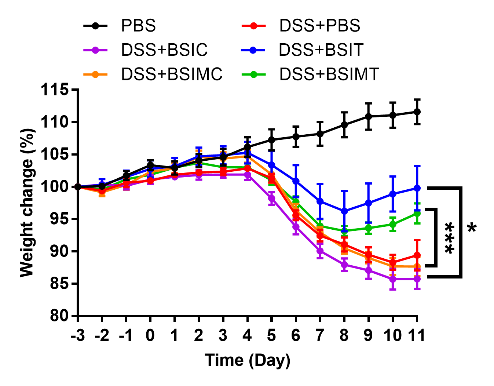


**Figure S5. Weight change of mice treated with therapeutic bacteria.**

Weight change of mice. Data are mean ± SEM, n = 10 for each group. Mice were weighed daily and their weights are presented as % change from day -3. Statistical analysis was performed using a paired *t* test; **p* < 0.05, ****p* < 0.001.


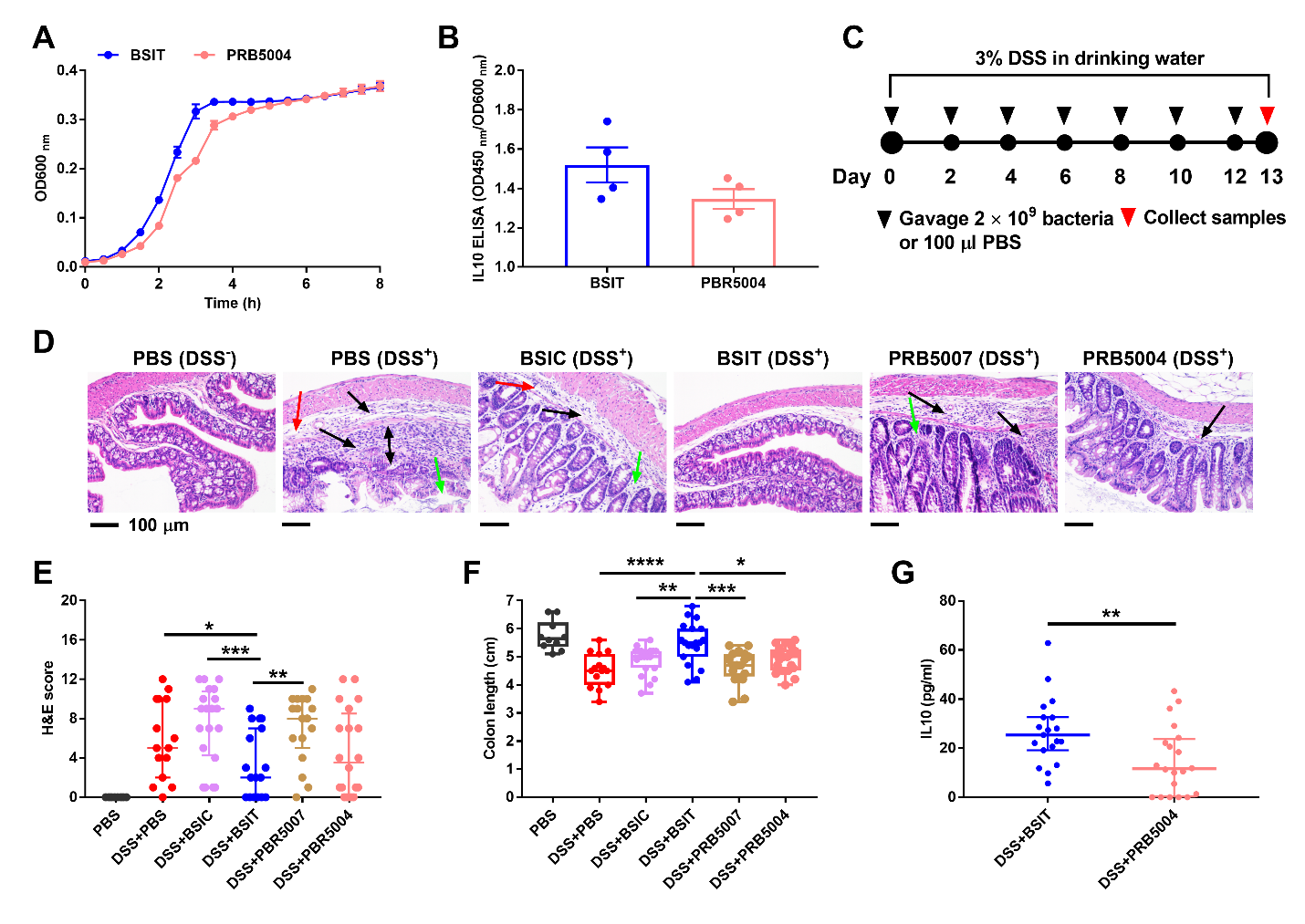


**Figure S6. Comparision of sensing and responding system with secIL10 continuous expression in ameliorating intestinal inflammation.**

**(A)** Growth profiles of secIL10 constitutive expression strain PRB5004. To achieve secIL10 constitutive expression, P*_ykg_* promoter of secIL10 in pBSIT was replaced with a constitutive expression promoter J23115, and the engineered plasmid was transformed into EcNΔ*asd*, resulting secIL10 continuous expression strain PRB5004. Strains were cultured in LB media, and OD_600nm_ was measured every 30 min for 8 h. For animal experiments, *yebF* constitutive expression (P_J23115_-*yebF*) strain PRB5007 was constructed as a control.

**(B)** Detection of secIL10 production in PRB5004. PRB5004 was cultured to the early stationary phase in LB media. The sensing and responding therapeutic strain BSIT was cultured to OD_600_ of 0.6 and then induced with 20 µM TPEN for 4 h in LB media. Following, the supernatants of cultures were collected and detected by IL10 ELISA. Data are mean ± SEM, n = 4 biological independent repeats.

**(C)** Experimental design of therapeutic bacteria applied to DSS induced gut inflammation. For the healthy control group (PBS treatment), n = 10 (5 male + 5 female). For all other groups, n = 20 (10 male + 10 female). Note: there is no pretreatment (pre-gavage of bacteria before DSS treatment) for mice.

**(D)** Representative hematoxylin and eosin (H&E) stained images of mouse distal colon sections from each experimental group. Black arrows: inflammatory cell infiltrates in the mucosa and submucosa. Double-headed black arrow: lamina propria expansion. Red arrows: loose of connective tissue. Green arrows: disappearance of glands and goblet cells.

**(E)** Histological score of colon tissue. Mouse distal colon tissue was collected, fixed, sliced, stained (H&E staining), and scored. Data are median ± interquartile range, showing all available points, and individual dots represent individual mice in each group. Statistical analysis was performed using Kruskal-Wallis test; **p* < 0.05, ***p* < 0.01, ****p* < 0.001.

**(F)** Colon length. Data are presented in a box & whiskers plot with min to max, showing all available points, and individual dots represent individual mice. Statistical analysis was performed using Kruskal-Wallis test; **p* < 0.05, ***p* < 0.01, ****p* < 0.001, *****p* < 0.0001.

**(G)** Concentration of secIL10 in mice serum. Mice serums were collected on day 13 and tested for secIL10 concentration by ELISA. Data are median ± interquartile range, showing all available points, and individual dots represent individual mice. Statistical analysis was performed using Mann-Whitney test; ***p* < 0.01.

**Supporting Tables**

**Table S2. Bacteria and plasmids utilized in this study**

| **Strains or plasmids** | **Genotype and function** | **Reference** |
| --- | --- | --- |
| **Strains** |  |  |
| BSI | Biosensor EcN harboring pBSI2 | This work |
| BSIM | Memory circuit biosensor EcN harboring pBSIM1 and pBSIM2 | This work |
| BSIT | EcNΔ*asd* harboring pBSIT | This work |
| BSIC | EcNΔ*asd* harboring pBSIC | This work |
| BSIMT | EcNΔ*asd* harboring pBSIMT and pBSIM1 | This work |
| BSIMC | EcNΔ*asd* harboring pBSIMC and pBSIM1 | This work |
| PRB5000 | Biosensor EcN harboring pBSI1 | This work |
| PRB5001 | Biosensor EcN harboring pBSIDZ1 | This work |
| PRB5002 | Biosensor EcN harboring pBSIDZ2 | This work |
| PRB5003 | EcNΔ*asd* harboring pBSIDZ3 | This work |
| PRB5004 | EcNΔ*asd* harboring pBSIDZ4 | This work |
| PRB5005 | EcNΔ*asd* harboring pBSIDZ5 | This work |
| PRB5006 | EcNΔ*asd* harboring pBSIDZ6 | This work |
| PRB5007 | EcNΔ*asd* harboring pBSIDZ7 | This work |
| *E. coli* DH5α | Cloning host | Lab stock |
| *Escherichia coli* Nissle 1917 (EcN) | Engineering strain | Lab stock |
| EcNΔ*asd* | EcN deleted *asd* essential gene | This work |
| *C. difficile* R20291 | Ribotype 027 strain, used for biosensor test in a CDI model | Lab stock |
| *C. difficile* CD196 | Ribotype 027 strain, used for biosensor test in a CDI model | Lab stock |
| **Plasmids** |  |  |
| pBSI1 | pColE1 containing P*_ykg_*-*sfgfp* | This work |
| pBSI2 | pColE1 containing P*_ykg_*-*sfgfp* and J23109-*zur* | This work |
| pBSIM1 | pColE1 containing P*_ykg_*-*int8* and J23109-*zur* | This work |
| pBSIM2 | p15A containing reversed *sfgfp* flanked by integrase 8 recognization sites *attB* and *attP* | This work |
| pBSIT | pColE1 containing P*_ykg_*-*secIL10*-P*_asd_*-*asd* and J23109-*zur* | This work |
| pBSIC | pColE1 containing P*_ykg_*-*yebF*-P*_asd_*-*asd* and J23109-*zur* | This work |
| pBSIMT | p15A containing reversed *secIL10* flanked by integrase 8 recognization sites *attB* and *attP* and P*_asd_*-*asd* | This work |
| pBSIMC | p15A containing reversed *yebF* flanked by integrase 8 recognization sites *attB* and *attP* and P*_asd_*-*asd* | This work |
| pBSIDZ1 | pColE1 containing P*_ykg_*-*yebF* and J23109-*zur* | This work |
| pBSIDZ2 | pColE1 containing P*_ykg_*-*secIL10* and J23109-*zur* | This work |
| pBSIDZ3 | pColE1 containing J23109-*secIL10*-P*_asd_*-*asd* and J23109-*zur* | This work |
| pBSIDZ4 | pColE1 containing J23115-*secIL10*-P*_asd_*-*asd* and J23109-*zur* | This work |
| pBSIDZ5 | pColE1 containing J23105-*secIL10*-P*_asd_*-*asd* and J23109-*zur* | This work |
| pBSIDZ6 | pColE1 containing J23108-*secIL10*-P*_asd_*-*asd* and J23109-*zur* | This work |
| pBSIDZ7 | pColE1 containing J23115-*yebF*-P*_asd_*-*asd* and J23109-*zur* | This work |
| pCas | plasmid with Cas9 gene | Lab stock |
| pTargetF | Plasmid used for sgRNA construction for *asd* gene deletion | Lab stock |

**Table S3. Primers utilized in this study**

| **Primer** | **Sequence (5’ to 3’)** | **PCR product** |
| --- | --- | --- |
|  |  |  |
| 1-F | AGGCCCTTTCGTCTTCACCTCGAGTAACGGCAATAAACTGTTCACTTCAG | P*_ykg_* for pBSI1 |
| 1-R | CTAGTATTTCTCCTCTTTCATTTTTACCTGTTATGTTATAACA |  |
| 2-F | TGAAAGAGGAGAAATACTAGATGCGTAAAGGCGAAGAGCTGTTCA | *sfgfp* for pBSI1 |
| 2-R | ATGCCTGGTCTAGATTATTATCATCATTTGTACAGTTCATCCATA |  |
| 3-F | GCCTTTTTAGTTAGAAAGCTTTAGCGAGGTTTCTTTTTCACCT | *zur* for pBSI2 |
| 3-R | GAATTCAGGAGATAATAT ATGGAAAAGACCACAACGCAGGAGT |  |
| 4-F | GCTAGCACAGTCCCTAGGACTGAGCTAGCTGTAAA | J23109 for pBSI2 |
| 4-R | ACGTCTCATTTTCGCCAGATATCGATTTACAGCTAGCTCAGTCCTAGGGACTGTGCTAGC |  |
| 5-F | TAAGGATGATTAATAATCTAGACCAGGCATCAAATAAAACGAAAGGCTCA | Linear pBSI1 without *sfgfp* for pBSIM1 |
| 5-R | AATCCTAGTTATTGTTTTGCTAGCCTTTACCTGTTATGTTATAACATAACC |  |
| 6-F | AGGCTAGCAAAACAATAACTAGGATTCGAATGAAAGTTGCCGTTTATTGTCGTG | *int8* for pBSIM1 |
| 6-R | ATGCCTGGTCTAGATTATTAATCATCCTTAGCGAAAGCTAAGGA |  |
| 7-F | TTGACAGCTAGCTCAGTCCTAGGTATAATGCTAGC | J23119 for pBSIM2 |
| 7-R | GCTAGCATTATACCTAGGACTGAGCTAGCTGTCAA |  |
| 8-F | TTGACAGCTAGCTCAGTCCTAGGTATAATGCTAGCAGTTCGATGAGAGCGATAACCAATCATCAGATAACTATGG | *attB-sfgfp*-*attP* for pBSIM2 |
| 8-R | TCGGGTGGGCCTTTCTGCGTTTATATTAATAAACTATGGAAGTATGTACA |  |
| 10/11-F | TAGCAAAACAATAACTAGGATTCGAGGAGAAAAACATGAAAAAAAGAGGGGCGTTTTTAG | *yebF* for pBSIDZ2 |
| 10-R | GAAAGCTAAGGATTTTTTTTATCTGTTAACGCCGCTGATATTCCGCCATTCCC |  |
| 11-R | CGTGCCTTGGCCCGGGCTACGCCGCTGATATTCCGCCA | *yebF* for pBSIDZ3 |
| 12-F | AGCCCGGGCCAAGGCACGCA | *IL10* for *yebF-IL10* fusion expression |
| 12-R | TAAGGATTTTTTTTATCTGTTAGTTGCGAATTTTCATGGTCATATACGCT |  |
| 13-F | CAGATAAAAAAAATCCTTAGCTTTC | Linear pBSI2 without *sfgfp* for pBSIDZ3 |
| 13-R | TCGAATCCTAGTTATTGTTTTGCTA |  |
| 14-F | GGATCCATAATCAGGATCAATAA | P*_asd_*-*asd* |
| 14-R | TTACGCCAGTTGACGAAGCATCCGA |  |
| 15-F | TGCTTCGTCAACTGGCGTAACAGATAAAAAAAATCCTTAGCTTTC | Linear pBSIDZ2 for pBSIC |
| 15-R | TTGATCCTGATTATGGATCC ACGCCGCTGATATTCCGCCATT |  |
| 16-F | TGATCCTGATTATGGATCCTTAGTTGCGAATTTTCATGGTCA | Linear pBSIDZ3 for pBSIT |
| 16-R | TTGATCCTGATTATGGATCC ACGCCGCTGATATTCCGCCATT |  |
| 17-F | TGCGCTCGGTCGTTCGGCTGCGGCGAGCGGTATCAGCTCACTCAA | For *attB*-*rsecIL10*-*attP* and *attB*-*ryebF*-*attP* amplification |
| 17-R | TCTGTTGTTTGTCGGTGAACGCTCTCTACTAGAGTCACACTGGCTCACCTTCG |  |
| 18-F | GAGAGCGTTCACCGACAAACAACAGA | Linear pMSIM1 for pBSIMT and pBSIMC |
| 18-R | CGCCGCAGCCGAACGACCGAGCGCAG |  |
| T-F | GGTATCAGCTCACTCAAAGG | *sfgfp* flipping detection primers |
| T-R | TGACATCACCATCCAGTTCC |  |
| *amp*-Q-F | GAAGATCAGTTGGGTGCACG | Reference gene for *sfgfp* flipping detection |
| *amp*-Q-R | TCACTCATGGTTATGGCAGC |  |
| *sfgfp*-Q-F | TGCGGTTTACCAGGGTATCG | *sfgfp* transcription analysis |
| *sfgfp*-Q-R | TATGTGCAGGAACGCACGAT |  |
| 16S-F | TAATACCTTTGCTCATTG | Reference gene for transcription analysis |
| 16S-R | CCAGTAATTCCGATTAAC |  |
